# Chronic caffeine treatment reverses memory impairment and the expression of brain BNDF and TrkB in the PS1/APP double transgenic mouse model of Alzheimer’s disease

**DOI:** 10.3892/mmr.2013.1601

**Published:** 2013-07-25

**Authors:** KUN HAN, NING JIA, JI LI, LI YANG, LIAN-QIU MIN

**Affiliations:** Department of Neurology, The First Affiliated Hospital of Liaoning Medical University, Jinzhou, Liaoning 121001, P.R. China

**Keywords:** caffeine, memory, brain neurotrophic derived factor, TrkB, Alzheimer’s disease

## Abstract

The objective of this study was to investigate the effects of varying doses of caffeine on memory impairment and the expression of brain neurotrophic derived factor (BNDF) and TrkB in PS1/APP double transgenic mouse models. PS1/APP double transgenic mice were administered 0.3 ml/day of saline, 1.5 mg/day of caffeine or 0.75 mg/day of caffeine for eight weeks. A water maze test and western blotting were used to determine the memory capability and expression of hippocampal BNDF and TrkB of the mice. The results demonstrated that 0.75 mg/day and 1.5 mg/day doses of caffeine significantly increased memory capability and the expression of hippocampal BDNF and TrkB in PS1/APP mice with a dose-response effect. The results suggested that chronic caffeine treatment may reverse memory impairment in PS1/APP transgenic mice, and BDNF and its receptor TrkB, may be involved in this process.

## Introduction

Alzheimer’s disease (AD) is a neurodegenerative disorder resulting in progressive cognitive impairment. It has been reported that AD is the most common form of dementia among older people and the worldwide prevalence of the disease is estimated at >24 million cases (1). Medical treatment for AD patients is placing an increasing burden on physicians and families every year. Clinically, there are a variety of drugs available for AD, such as cholinesterase inhibitors, glutamate receptor antagonist and free radical scavengers. However, these drugs cannot target the pathogenesis of the disease closely and have significant side-effects (2). Therefore, it is important to find a new type of drug and to clarify the mechanism of AD pathophysiology. Caffeine is one of the most widely consumed psychoactive substances in the world (3). Recently, studies have demonstrated that caffeine intake may reduce the cognitive impairment in elderly patients and the risk of AD in later life (4,5). It also has been revealed that AD patients consume markedly less caffeine than people without AD (6). Elevated levels of β-amyloid (Aβ) in the brain and progressive cognitive impairment are the main characteristics of AD. Several studies have indicated that caffeine intake (1.5 mg/day) may reverse cognitive impairment and decrease brain Aβ levels in aged AD mice (7,8).

Brain neurotrophic derived factor (BDNF), a member of the neurotrophin family, is essential for growth, survival and the differentiation of neurons. Furthermore, BDNF is involved in learning and memory by binding to its main functional receptor (TrkB), in the hippocampus, cortex and basal forebrain (9). The levels of BDNF and TrkB have been reported to be lower in AD patients (10,11). It has been demonstrated that BDNF signaling, through TrkB, is involved in the pathophysiology and cognitive deficits of AD (12). PS1/APP double transgenic mice expressing the human APPswe and PS1-A246E mutations are a widely used AD model which may imitate the main pathophysiology process of AD. The present study was conducted in order to investigate the effect of varying caffeine doses on memory impairment and the expression of brain BNDF and TrkB in PS1/APP double transgenic mice.

## Materials and methods

### Drugs

Caffeine (lot number, 1001176428) was purchased from Sigma Corporation (St. Louis, MO, USA).

### Animals

PS1/APP double transgenic mice (genetic background C57BL/6J), containing the human APPswe and PS1-A246E mutations, were obtained from the Institute of Laboratory Animals at the Chinese Academy of Medical Sciences (Beijing, China). Wild-type C57/BL6J mice were used as controls. All mice were housed in the Laboratory Animal Center of Liaoning Medical University (Jinzhou, Liaoning, China). All mice were maintained in an air-conditioned room with a 12-h light and 12-h dark cycle, fed a standard diet and water was available *ad libitum*. The ethical approval for this study was obtained from the Ethics Committee of the Liaoning Medical University.

### Caffeine treatment

In this study, 24 PS1/APP double transgenic mice were randomly divided into three groups (age, 24 months; n=8) and 0.3 ml/day of saline (Tg-control), 1.5 mg/day of caffeine (Caff-H) and 0.75 mg/day of caffeine (Caff-L) were administered into the stomachs of mice in the three groups, respectively. Furthermore, eight wild-type C57/BL6J (NT) mice were administered 0.3 ml/day of saline at the same time (WT). All mice were treated for eight weeks.

### Water maze

At the end of the seventh week of the experiment, a water maze experiment was performed in order to evaluate the spatial reference memory of the mice. We used a circular inflatable pool (diameter 120 cm; height 90 cm) filled with opaque water, containing a submerged escape platform (diameter, 9 cm) 2.0 cm below the surface of the water. The water was maintained at a constant temperature throughout the experiment (25±0.5°C) and the pool was divided into four equal quadrants by black lines drawn on the floor of the pool. The experiment was divided into place navigation and spatial probe tests. The place navigation test lasted for four days. During this test, mice were randomly placed into one of the four equal quadrants of the pool. Mice were allowed to search for the platform for 60 sec. If the mice did not locate the platform within 60 sec, they were guided to it and left on the platform for 10 sec. The latency time (the time from entering the water to standing on the platform) was recorded. The spatial probe test was performed on the fifth day of the experiment. The platform was removed from the pool and the mice were placed into the water at any location. The mice were allowed to swim in the maze for 60 sec. Swimming time, the time that the mice spent in target quadrant (where the platform was), was recorded.

### Western blotting

At the end of the experiment, the mice were decapitated and the brains were rapidly removed on ice, the hippocampus was quickly dissected and stored at −70°C until required. The hippocampal tissue was homogenized in a lysis buffer (50 mmol/l Tris-HCl, 5 mmol/l EDTA, 1% sodium deoxycholate, 150 mmol/l NaCl, 0.5% Triton X-100, 500 μmol/l Na_3_VO_4_, 10 μmol/l AEBSF, 10 mmol/l NaF). The homogenates were subsequently centrifuged at 12,000 × g for 10 min at 4°C, and the supernatants were collected for protein concentration determination using a protein assay (Bio-Rad, Hercules, CA, USA). Equal amounts of protein extract were added to 8% SDS-polyacrylamide gel and transferred onto a PVDF membrane. Western blotting was performed using rabbit anti-BDNF and anti-TrkB [1:1,000, SC546, Santa Cruz Biotechnology, Inc. (Santa Cruz, CA, USA); 1:1,000, ab38306, Abcam (Cambridge, MA, USA)] followed by a chemiluminescence substrate (32109, ECL Plus, Amersham; kit contents: luminol/enhancer, 25 ml, stable peroxide buffer, 25 ml) and quantified. The BDNF and TrkB antisera detected the distinct mature form of BDNF (mBDNF) and TrkB bands as described previously (13). Recombinant human BDNF and TrkB (BioVision, Mountain View, CA, USA) were added to act as a positive control. The blots were stripped and reprobed with anti-actin (1:4,000) to control loading variations. Quantity One software (Bio-Rad) was used to quantify the protein bands. The results were expressed as the means ± SEM of the ratio of immunoreactivity normalized by β-actin.

### Statistical analysis

Statistical analysis was performed using the Student’s t-test and one-way ANOVA followed by Newman-Keuls multiple comparisons test. P<0.05 was considered to indicate a statistically significant difference.

## Results

**Figure 1:**
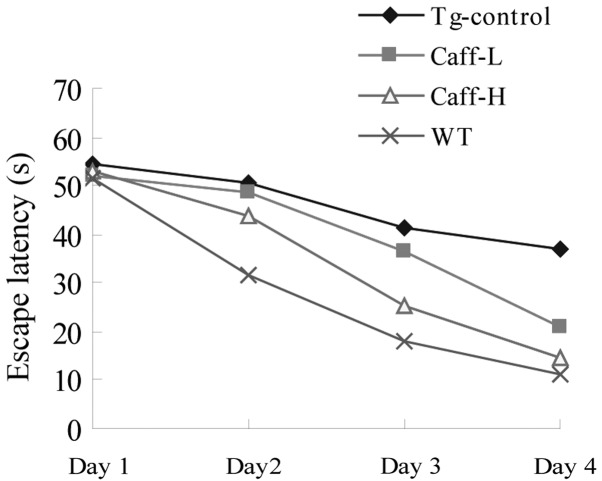
Effect of caffeine treatment on the escape latency time of PS1/APP and wild-type mice in the hidden platform acquisition test of the water maze. Caff-H, 1.5 mg/day of caffeine; Caff-L, 0.75 mg/day of caffeine; WT, 0.3 ml/day of saline.

**Figure 2:**
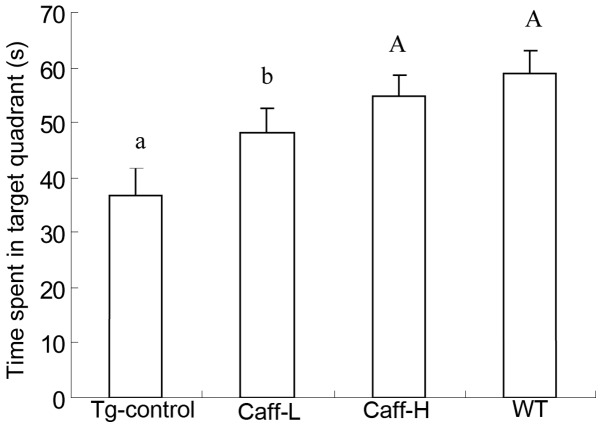
Effect of caffeine treatment on the time that PS1/APP and wild-type mice spent in the target quadrant during the probe trial of the water maze. Graphic represents means ± SEM of the time spent in the target quadrant for the different groups. b or A indicate significant differences at P<0.05 and P<0.01 levels, respectively, compared to Tg-control, a. Caff-H, 1.5 mg/day of caffeine; Caff-L, 0.75 mg/day of caffeine; WT, 0.3 ml/day of saline.

In order to investigate the effect of caffeine treatment on spatial learning and memory of PS1/APP double transgenic mice, a water maze test was performed. The results are shown in Figs. 1 and 2. As shown in Fig. 1, the Tg-control mice took significantly longer to locate the platform than the WT mice (P<0.01), suggesting that the ability for spatial learning had decreased in the AD model mice. Administering varying doses of caffeine may significantly decrease the escape latency time of PS1/APP mice (P<0.01). The escape latency time of Caff-H mice was significantly higher compared with that of the Caff-L mice indicating that there is a dose-response effect for caffeine treatment. For the spatial probe test (Fig. 2), the results demonstrated that caffeine treatment could significantly decrease the time that the PS1/APP mice spent in the target quadrant (P<0.01), and Caff-H mice spent longer in the target quadrant than Caff-L mice (P<0.05). The water maze test revealed that caffeine treatment was capable of significantly increasing the ability of spatial learning and memory in PS1/APP mice with a dose-response effect.

**Figure 3:**
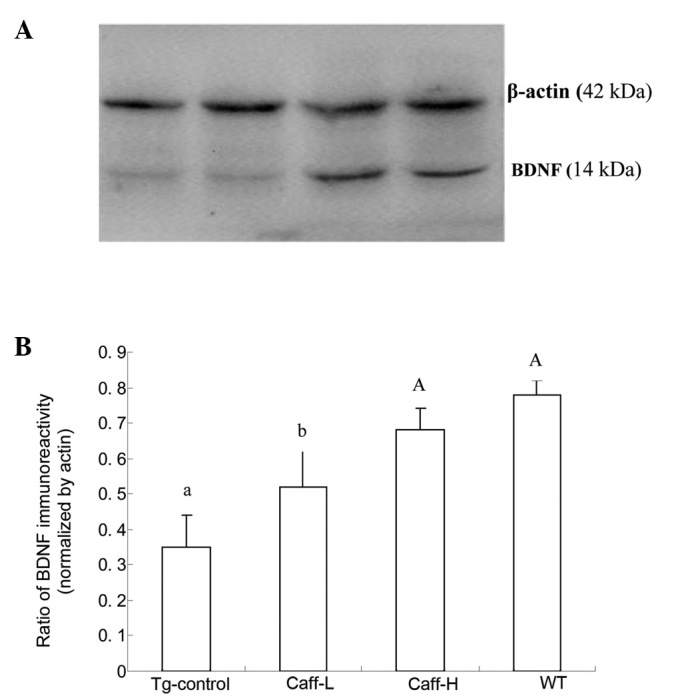
Effect of caffeine treatment on the expression of BDNF in PS1/APP and wild-type mice by western blotting. (A) Representative bands: BDNF at 14 kDa; β-actin at 42 kDa. (B) Graphic represents the means ± SEM of the ratio of immunoreactivity of BDNF in the different groups. Values with b indicate significant differences at P<0.05, and values with A indicate significant differences at P<0.01 levels, compared to Tg-control, a. Caff-H, 1.5 mg/day of caffeine; Caff-L, 0.75 mg/day of caffeine; WT, 0.3 ml/day of saline. BDNF, hippocampal brain neurotrophic derived factor.

**Figure 4:**
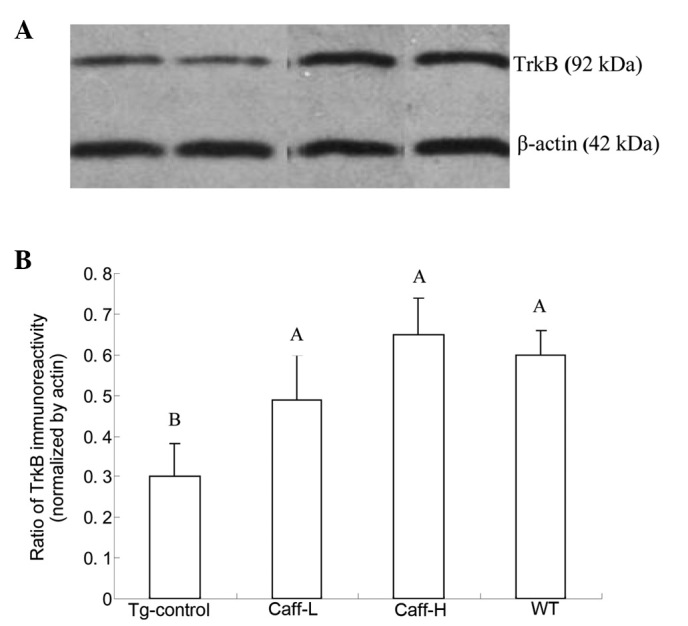
Effect of caffeine treatment on the expression of hippocampal TrkB in PS1/APP and wild-type mice by western blotting. (A) Representative bands: TrkB at 92 kDa; β-actin at 42 kDa. (B) Graphic represents the means ± SEM of the ratio of immunoreactivity of TrkB in the different groups. Values with A indicate significant differences at P<0.01, compared to Tg-control, B. Caff-H, 1.5 mg/day of caffeine; Caff-L, 0.75 mg/day of caffeine; WT, 0.3 ml/day of saline.

In order to determine the effect of caffeine treatments on the expression of hippocampal BDNF and TrkB in mice, western blotting was performed. The results are shown in Figs. 3 and 4. The expression of hippocampal BDNF and TrkB of PS1/APP mice treated with saline was significantly lower compared with that of WT mice (P<0.01). It was also demonstrated that caffeine treatment significantly increased the expression of hippocampal BDNF and TrkB, and a high dose caffeine treatment obtained a higher level of expression than a low dose of caffeine (P<0.05).

## Discussion

Several studies have demonstrated that caffeine intake (1.5 mg/day) is capable of reversing cognitive impairment in AD mice (7,8). As the effect of different doses of caffeine on cognitive impairment and the expression of hippocampal BDNF and TrkB in PS1/APP mice have been poorly investigated, the present study was conducted. The results demonstrated that low (0.75 mg/day) and high (1.5 mg/day) doses of caffeine increased spatial learning ability and the memory and expression of hippocampal BDNF and TrkB in PS1/APP mice with a dose-response effect.

It had been shown that the cognitive ability of AD mice decreased and 1.5 mg/day of caffeine was capable of reversing the cognitive impairment (7,8). The present study not only confirmed previous studies but also investigated the effect of low doses of caffeine (0.75 mg/day) on the cognitive impairment of AD mice. The results revealed that 0.75 mg/day of caffeine for eight weeks was capable of increasing spatial learning ability and memory in 12 month old PS1/APP transgenic mice. It has been reported that the oral administration of 3 mg/day of caffeine for two weeks was capable of improving cognitive impairment of 9.5 month old PS1/APP double transgenic mice (14). A previous study has demonstrated that 0.5 mg/day of caffeine in drinking water reduced the cholesterol-induced increase in Aβ and phosphorylated τ, which suggests that even particularly low doses of caffeine may protect against sporadic AD-like pathology (15). The varying doses of caffeine treatments in different studies may be caused by differences in the ages of mice or treatment time.

Studies have revealed that caffeine intake may reverse memory impairment and decrease the levels of Aβ in the brains of AD mice (7,8,14,16); however, the exact mechanism for the role of caffeine in memory impairment is unclear. Previous studies have indicated that the mechanism may be complex and involve a variety of aspects of memory ability. Long-term caffeine administration may improve memory by reducing the levels of Aβ through the suppression of the Aβ-producing enzymes, β- and γ-secretase (7,17). In another study, it has been demonstrated that caffeine is capable of decreasing the expression of pro-apoptotic phospho-JNK and phospho-ERK in the striatum and cortex, and stimulated PKA signaling in the striatum of APPswe mice. BDNF is crucial in neuronal plasticity, learning and memory. The levels of BDNF, and its main receptor TrkB, have been reported to decrease in AD. We hypothesized that BDNF and its receptor may be involved in the protective role of caffeine against memory impairment. Results of the present study have demonstrated that caffeine intake significantly increased the expression of BDNF, and its main receptor TrkB, in the brain, which is in agreement with our hypothesis. There is evidence to support our results. It has been demonstrated that BDNF, and TrkB, are capable of protecting against memory impairment and regulate neurogenesis in the hippocampus of AD (18). A recent study also supports the role of BDNF signaling through TrkB in the pathophysiology and cognitive deficits of AD (12). However, the exact mechanism of BDNF and its receptor involving caffeine in AD remains unclear and requires further investigation.

In conclusion, the present study reveals that 0.75 mg/day and 1.5 mg/day of caffeine for eight weeks is capable of reversing memory impairment in 12 month old PS1/APP transgenic mice, and BDNF and its receptor TrkB may be involved in the protective role of caffeine against memory impairment in AD.
